# Supplementary material for: Regulation Awareness and Experience of Additional Monitoring among Healthcare Professionals in Finland
Source: Healthcare (Basel). 2021 Nov 11;9(11):1540. doi: 10.3390/healthcare9111540 (PMC8622083; doi:10.3390/healthcare9111540)
Supplement: Supplementary file 1 [file healthcare-09-01540-s001.zip › healthcare-1436463-supplementary.pdf]

Dear Healthcare professional,

We invite you to complete a questionnaire concerning adverse event reporting of medicines.

Adverse event reporting is crucial in establishing a comprehensive safety profile of a medicine. The aim of this research is to examine the adverse event reporting knowledge and work methods of Finnish healthcare professionals. The results of this research will be published in a scientific journal and will be utilized in the planning of future educational events.

This questionnaire is anonymous and no personal information is collected. Only essential information will be collected and stored on a password protected server of University of Helsinki.

The questionnaire consists of 26 questions some of which are multiple-choice questions and some open-ended questions. It takes approximately 10-15 minutes to answer the questionnaire. The questions focus on your current opinions and beliefs. You do not need to seek information to answer. You can discontinue answering any time you want before submitting the answers at the end of the questionnaire.

Your trade or area union has sent this questionnaire to their members on behalf of the research group. Your contact details have not been shared with the researchers. **Answering the questionnaire is voluntary and by answering you are giving your informed consent to participate in the research.**

This investigator-driven research is a part of my pharmacy specialization studies at University of Helsinki and completely independent of activities performed by pharmaceutical companies (including my current employer Takeda Oy). Please contact me in case of questions concerning the questionnaire or the research.

Please submit your answers by 30-Jun-2019 in case you decide to participate in the research.

Yours faithfully,

Andreas Sandberg
University of Helsinki
andreas.sandberg@helsinki.fi

Background information

1. What is your schooling
    a. Physician
    b. Specialist physician
    c. Nurse
    d. Practical nurse
    e. Master of Pharmacy
    f. Bachelor of Pharmacy
    g. Other, what?

2. How many years have you been in your profession?
    a. Under 5 years
    b. 5 - 9 years
    c. 10 - 19 years
    d. Over 20 years

3. What is your primary workplace?
    a. Retail pharmacy
    b. Hospital pharmacy
    c. Hospital
    d. Healthcare center
    e. Private clinic
    f. Nursing home
    g. Pharmaceutical industry
    h. Government
    i. Other, what?

Adverse event reporting of medicines

4. How many times have you reported an adverse event to the local health authority (Fimea) or to the marketing authorization holder?
    a. I have not reported any adverse events
    b. 1-5 times
    c. 6-10 times
    d. Over 10 times

5. Which of the following claims are correct (there can be more than one right answer)?
    a. Healthcare professionals are encouraged to report adverse events even if uncertain medicine is the culprit
    b. Healthcare professionals are encouraged to report adverse events even if they do not have all the details of the event (e.g. patient details)
    c. All serious adverse events are known once the medicine enters the market
    d. Adverse events reported by Finnish healthcare professionals are handled locally and do not influence safety information in other countries
    e. Patients themselves can report adverse events to authorities or marketing authorization holders
    f. Healthcare professionals are also encouraged to report overdoses, misuse and medication errors
    g. Healthcare professionals are encouraged to report medicine use during pregnancy
    h. Healthcare professionals should report adverse events to the local health authority (Fimea) not the marketing authorization holder
    i. I do not know

6. Do you have enough information on how adverse events should be reported?
    a. Yes
    b. No

7. What information or tools would you need to make adverse event reporting easier?

Medicines under additional monitoring

8. Did you know that some medicines are under additional monitoring before answering this questionnaire?
    a. Yes
    b. No

9. Why are some medicines additionally monitored (there can be more than one right answer)?
    a. These medicines have more serious adverse events
    b. Adverse events are more common with these medicines
    c. Safety information has not yet been collected as much as desired for these medicines
    d. No reason. All medicines will be additionally monitored after the transition period
    e. I do not know

10. Did you know that medicines under additional monitoring must have a black triangle (▼) in the Summary of Product Characteristics/Package Leaflet/marketing materials before answering this questionnaire?
    a. Yes
    b. No

11. Has this black triangle (▼) been easy to notice?
    a. Never
    b. Seldom
    c. Sometimes

d. Often
e. Always

12. How do you react to medicines with a black triangle (▼) (e.g. positive, neutral, negative)? Why?

13. Do you know which medicines are under additional monitoring when prescribing/dispensing/administering medicines?
    a. Never
    b. Seldom
    c. Sometimes
    d. Often
    e. Always
    f. I do not work with patients

14. Where would you seek information to check if a medicine is under additional monitoring?

15. Do you report more readily adverse events for medicines under additional monitoring?
    a. Yes
    b. No

16. Why do you report/not report adverse events more readily for medicines under additional monitoring?

17. How could adverse event reporting be enhanced/developed for medicines under additional monitoring?

18. Are you more cautious when prescribing/dispensing/administering medicines under additional monitoring?
    a. Never
    b. Seldom
    c. Sometimes
    d. Often
    e. Always
    f. I do not work with patients

19. Why are/aren´t you more cautious when prescribing/dispensing/administering medicines under additional monitoring?

20. Do you tell the patient about additional monitoring?
    a. Never
    b. Seldom
    c. Sometimes
    d. Often
    e. Always
    f. I do not work with patients

21. Why do/don´t you tell the patient about additional monitoring?

22. Have patients asked you about additional monitoring/black triangle (▼)?

   a. Yes
   b. No

23. What kind of questions have you received?

24. Which parties have organized you training about adverse event reporting?
    a. I have not received training about adverse events reporting
    b. Outside service provider
    c. Internal trainer at the establishment
    d. Local health authority (Fimea)
    e. Other, what?

25. Which parties have organized you training about additional monitoring?
    a. I have not received training about additional monitoring
    b. Outside service provider
    c. Internal trainer at the establishment
    d. Local health authority (Fimea)
    e. Other, what?

26. Other comments and remarks of this questionnaire/additional monitoring/adverse event reporting?

Thank you for your answers!
